# Supplementary material for: Synthesis and preliminary PET imaging of 11C and 18F isotopologues of the ROS1/ALK inhibitor lorlatinib
Source: Nat Commun. 2017 Jun 8;8:15761. doi: 10.1038/ncomms15761 (PMC5472746; doi:10.1038/ncomms15761)
Supplement: Supplementary Information — Supplementary figures, supplementary table, supplementary discussion and supplementary references. [file ncomms15761-s1.pdf]

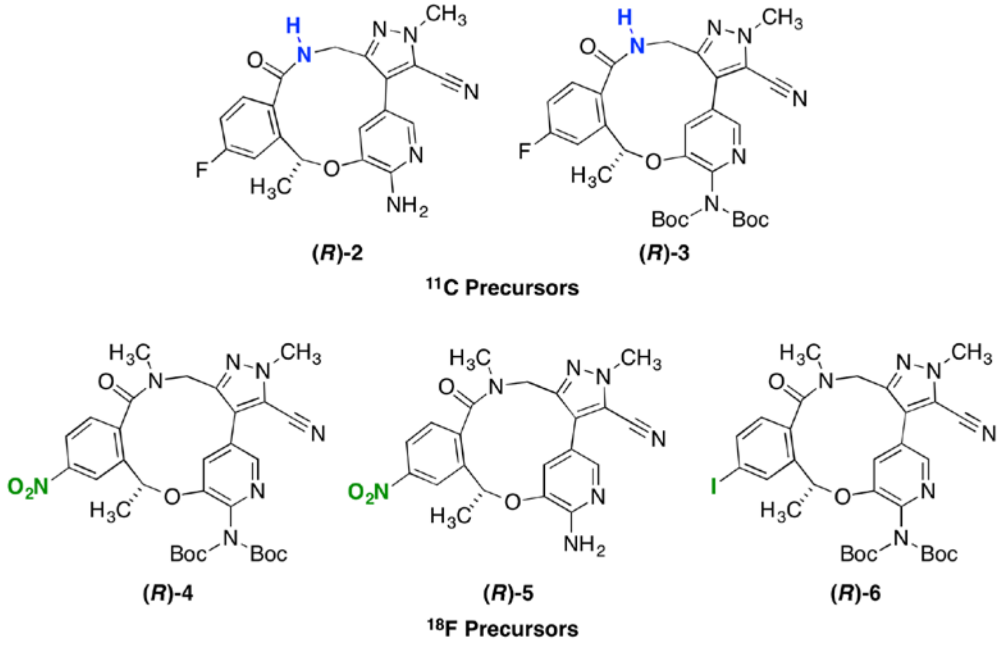

Supplementary Figure 1. Planned Precursors for Radiolabelling Studies

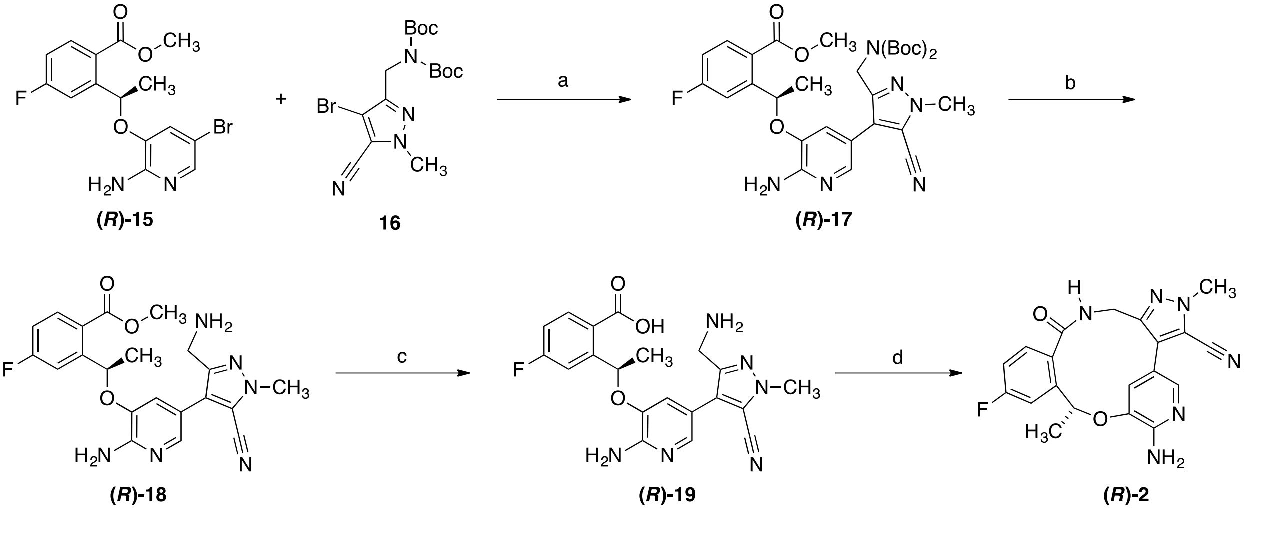

Supplementary Figure 2. Synthesis of ^{11}C-Labelling Precursor, ***(R)*-2**

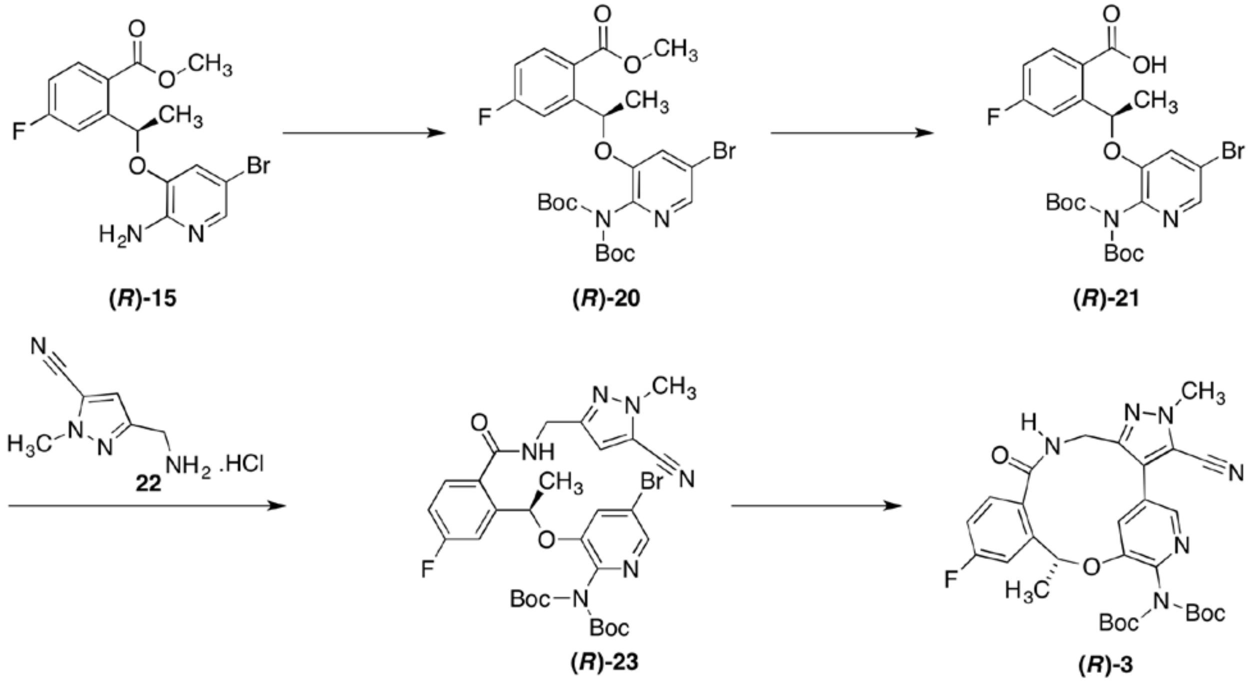

Supplementary Figure 3. Synthesis of Protected ^{11}C-Labelling Precursor, ***(R)*-3**

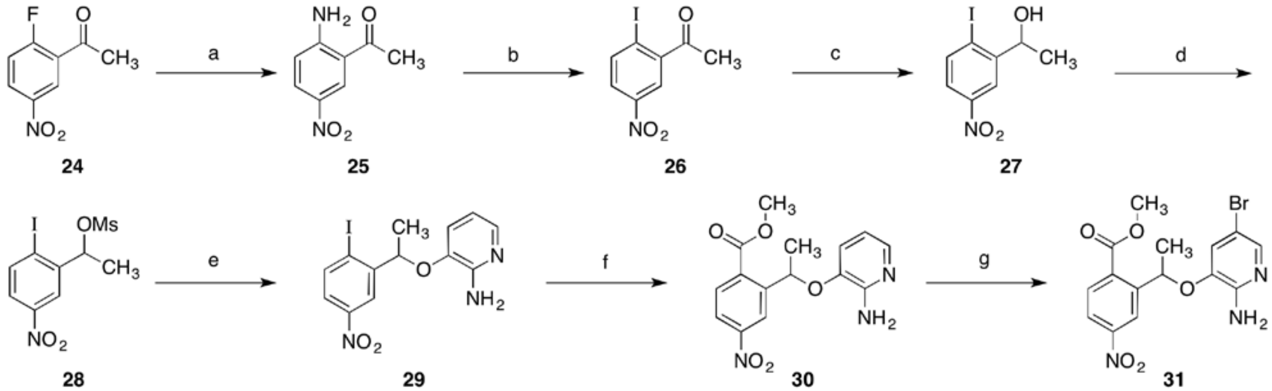

Supplementary Figure 4. Synthesis of Nitro-bearing Headgroup, **31**

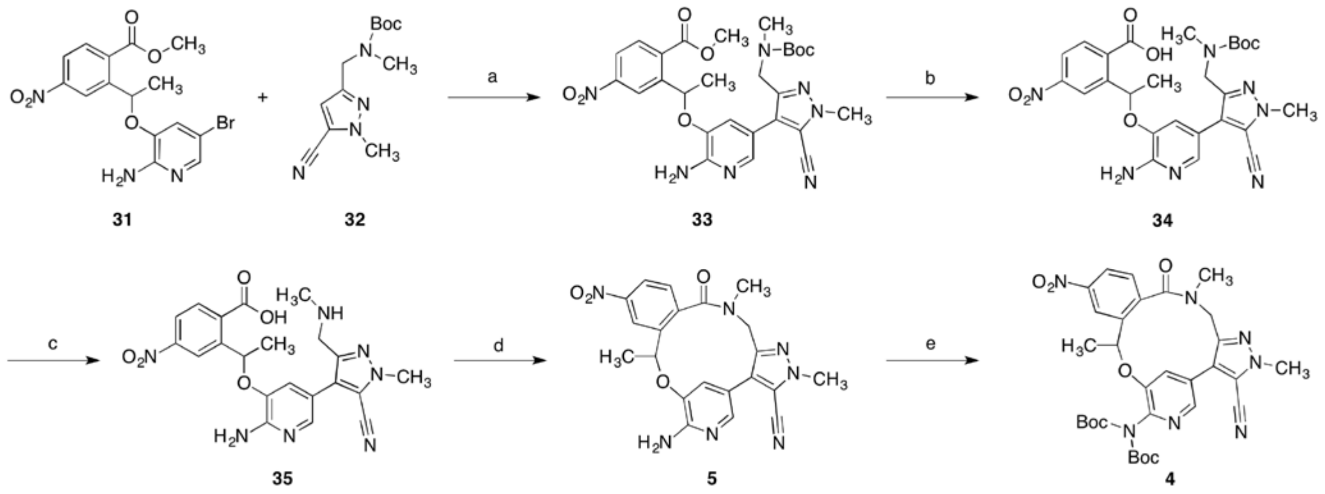

Supplementary Figure 5. Synthesis of F-18 Labelling Precursors, **4** and **5**

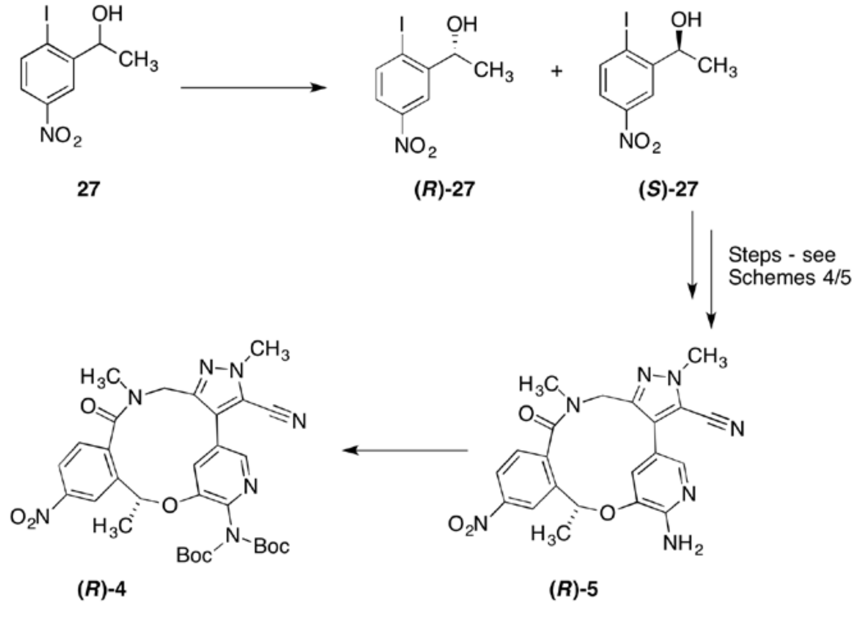

Supplementary Figure 6. Chiral Separation of (1*R*)-1-(2-iodo-5-nitrophenyl)ethanol ***(R)*-27**, and (1*S*)-1-(2-iodo-5-nitrophenyl)ethanol ***(S)*-27**

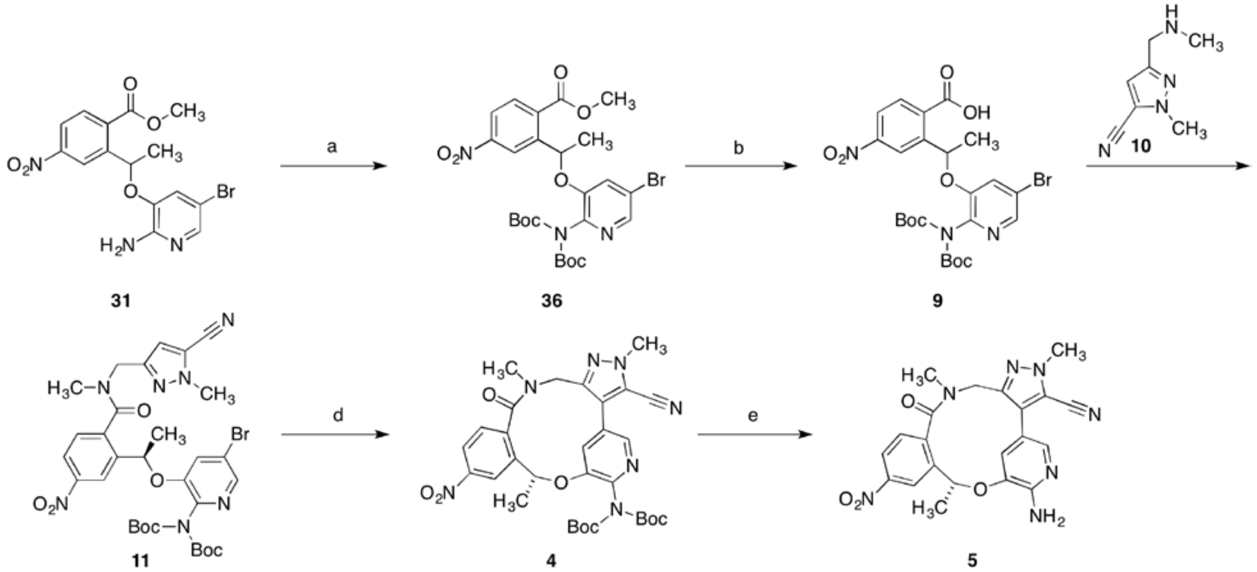

Supplementary Figure 7. Direct Arylation Approach to **4** and **5**

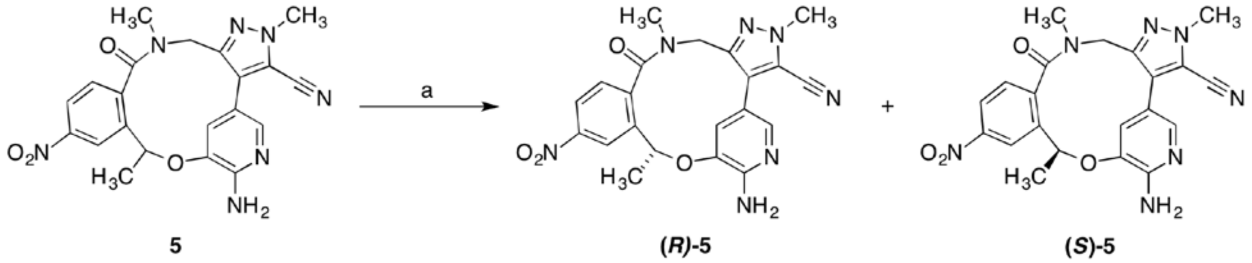

Supplementary Figure 8. Chiral Separation of macrocycles **(*R*)-5** and **(*S*)-5**

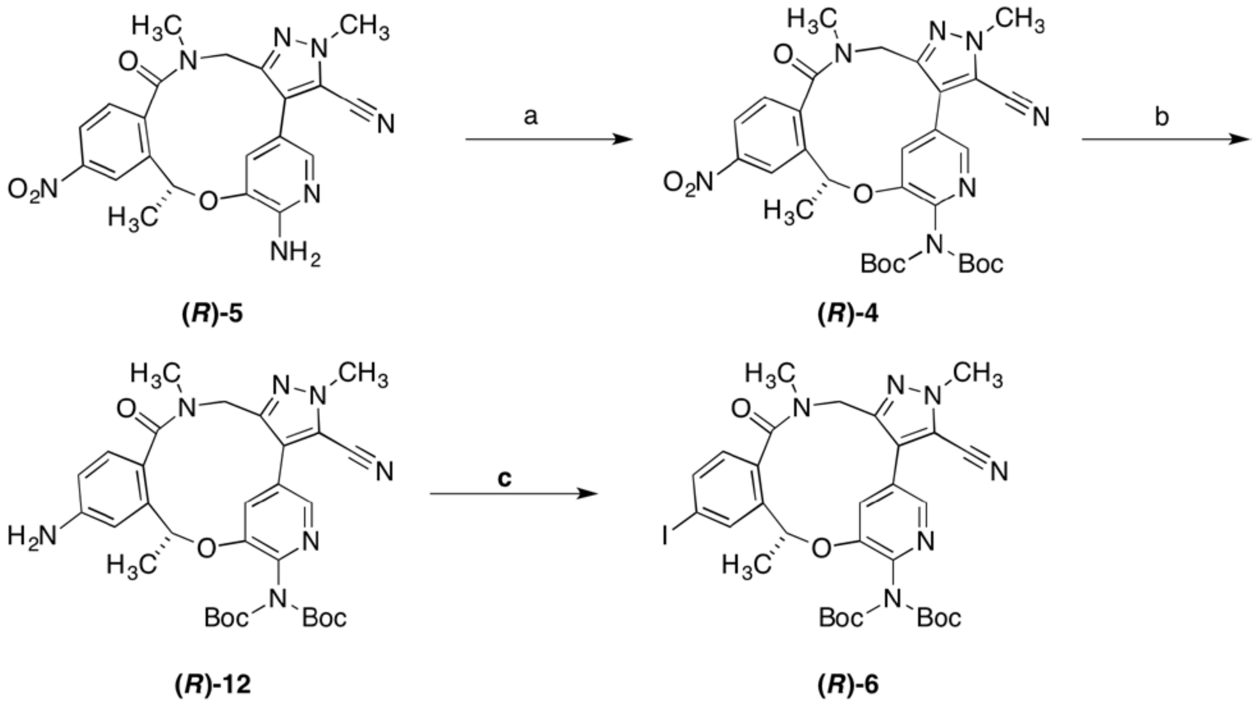

Supplementary Figure 9. Synthesis of F-18 Labelling Precursor, **(*R*)-6**

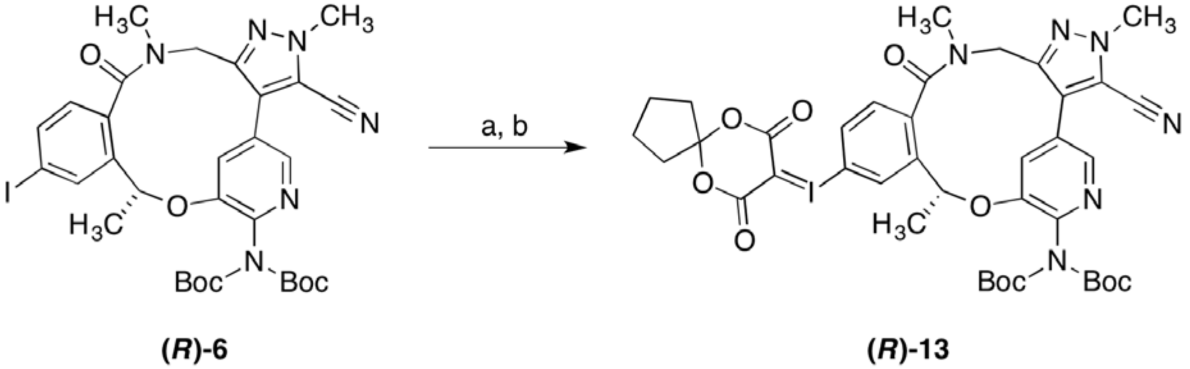

Supplementary Figure 10. Synthesis of ^{18}F-Labelling Precursor, **(*R*)-13**

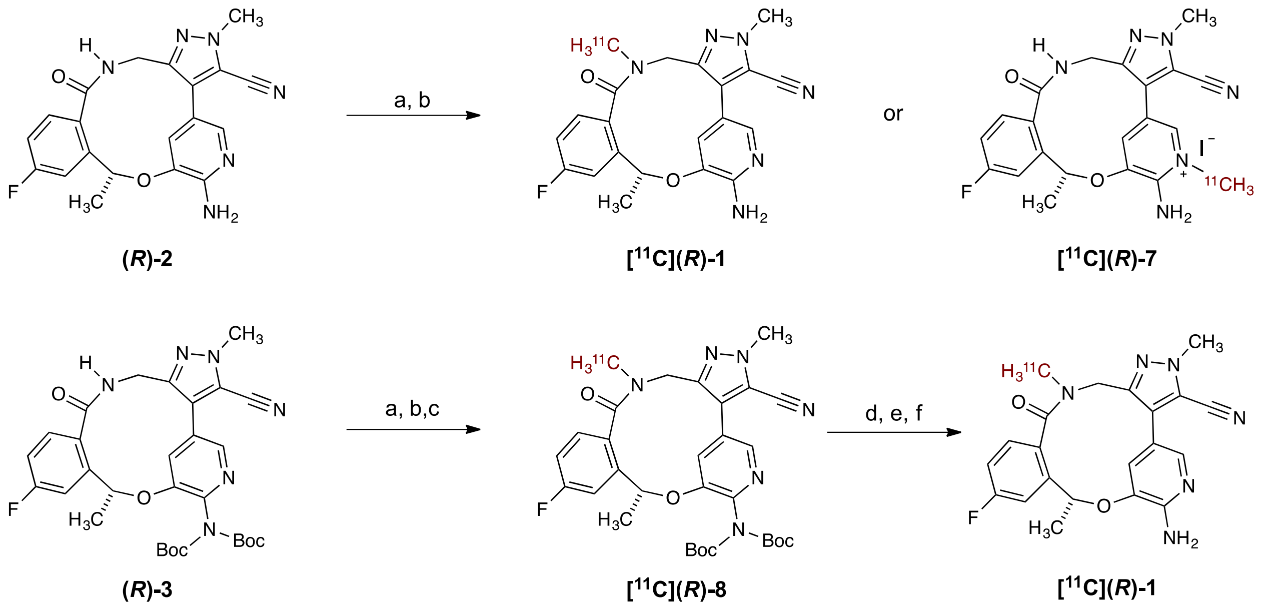

Supplementary Figure 11. One- and two-step syntheses of [^{11}C]lorlatinib ([^{11}C]***(R)*-1**)

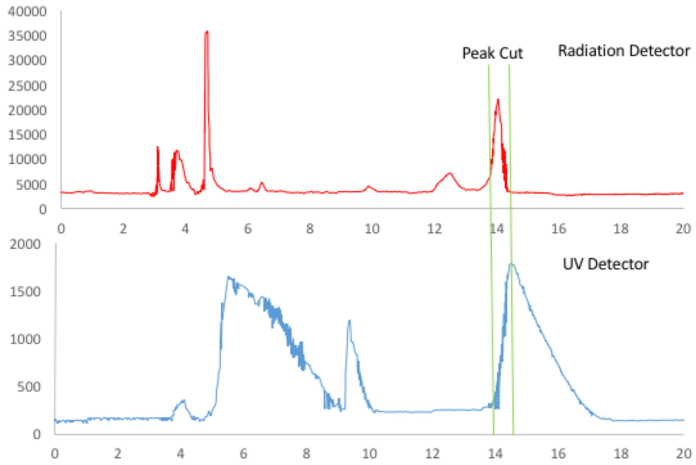

HPLC Column: Phenomenex Luna C-18 semiprep, 10 X 250 mm, 10 µm
HPLC Flow rate: 5 ml/min,
HPLC Mobile Phase: ACN:0.1M AMF (40:60)

Supplementary Figure 12. Semi-prep purification of 1-step methylation to prepare [^{11}C]lorlatinib.

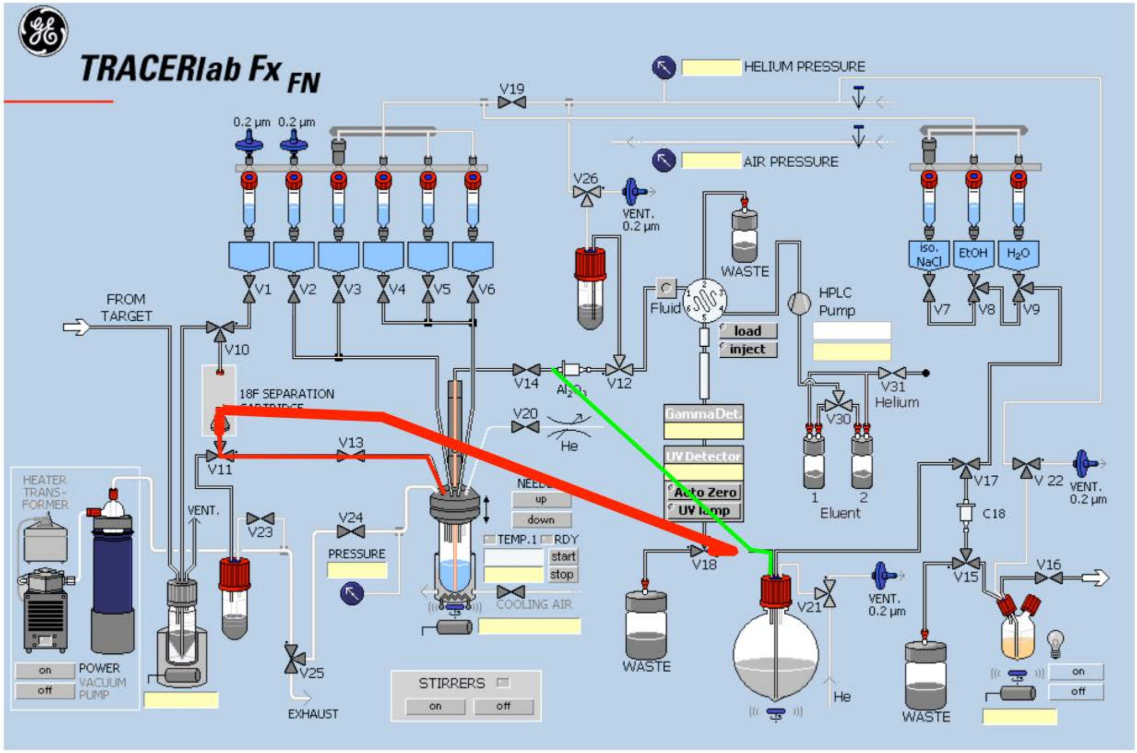

Supplementary Figure 13. Modified flow path of GE TracerLab FX_{FN}

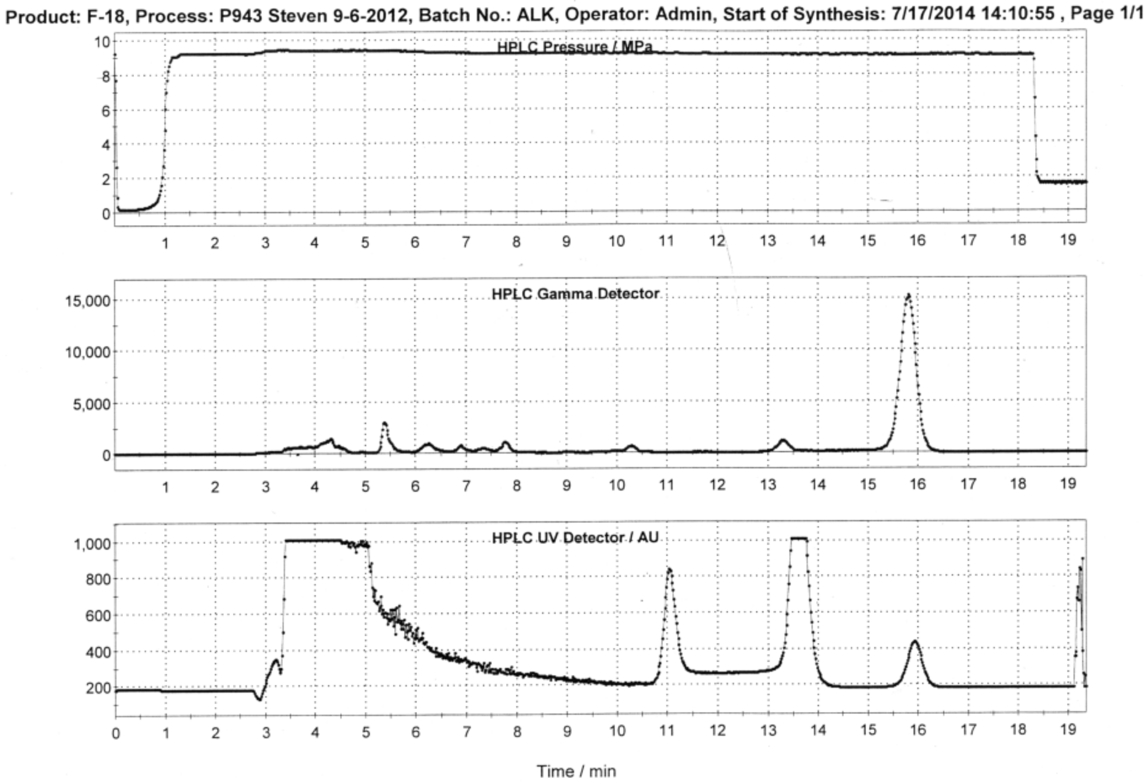

Supplementary Figure 14. Semi-prep purification of labeled protected intermediate on GE TracerLab $FX_{FN.}$ (HPLC Column: Phenomenex Luna C-18 semiprep, 10 X 250 mm, 10 µm; Flow rate: 5 ml/min; Mobile Phase: ACN:0.1M AMF (60:40)).

a) UV HPLC trace of QC sample

Waters
Project Name: test1
Reported by User: System

Breeze

SAMPLE INFORMATION

Sample Name:	C-11 ALK 102114	Acquired By:	System
Sample Type:	Unknow n	Date Acquired:	10/21/2014 1:44:47 PM
Vial:	1	Acq. Method:	ALK
Injection #:	1	Date Processed:	10/21/2014 2:04:59 PM
Injection Volume:	20.00 ul	Channel Name:	2487Channel 1
Run Time:	20.00 Minutes	Channel Desc.:	
Column Type:		Sample Set Name:	C11 ALK 102114

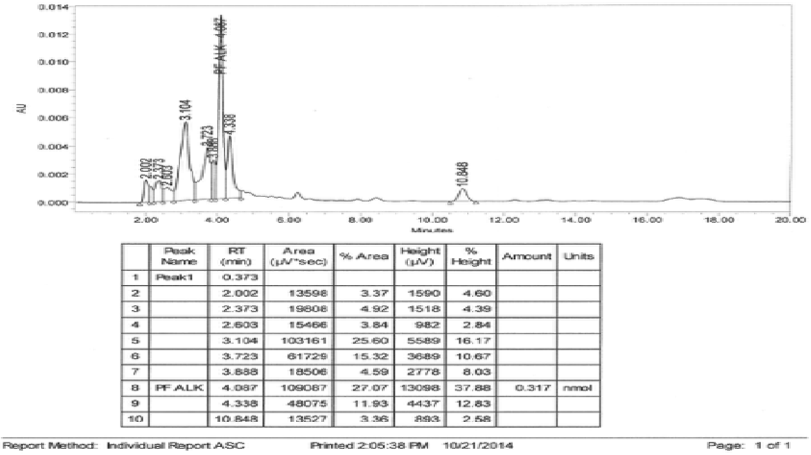

	Peak Name	RT (min)	Area (µV*sec)	% Area	Height (µV)	% Height	Amount	Units
1	Peak1	0.373						
2		2.002	13598	3.37	1590	4.60		
3		2.373	19808	4.92	1518	4.39		
4		2.603	15466	3.84	982	2.84		
5		3.104	103161	25.60	5589	16.17		
6		3.723	61729	15.32	3689	10.67		
7		3.888	18506	4.59	2778	8.03		
8	PF ALK	4.087	109087	27.07	13098	37.88	0.317	nmol
9		4.338	48075	11.93	4437	12.83		
10		10.848	13527	3.36	893	2.58		

Report Method: Individual Report ASC Printed 2:05:38 PM 10/21/2014 Page: 1 of 1

b) Radiation detector HPLC trace of QC sample

Waters
Project Name: test1
Reported by User: System

Breeze

SAMPLE INFORMATION

Sample Name:	C-11 ALK 102114	Acquired By:	System
Sample Type:	Unknow n	Date Acquired:	10/21/2014 1:44:47 PM
Vial:	1	Acq. Method:	ALK
Injection #:	1	Date Processed:	10/21/2014 2:04:59 PM
Injection Volume:	20.00 ul	Channel Name:	SATIN
Run Time:	20.00 Minutes	Channel Desc.:	
Column Type:		Sample Set Name:	C11 ALK 102114

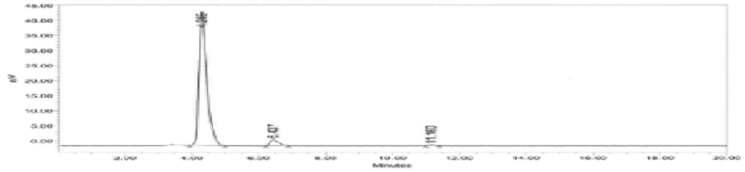

	RT (min)	Area (µV*sec)	% Area	Height (µV)	% Height
1	4.289	702276	95.17	44527	95.22
2	6.437	31908	4.32	1961	4.10
3	11.160	3719	0.50	273	0.58

Report Method: Individual Report ASC Printed 2:07:50 PM 10/21/2014 Page: 1 of 1

c) UV HPLC trace of QC sample with Standard added to confirm compound

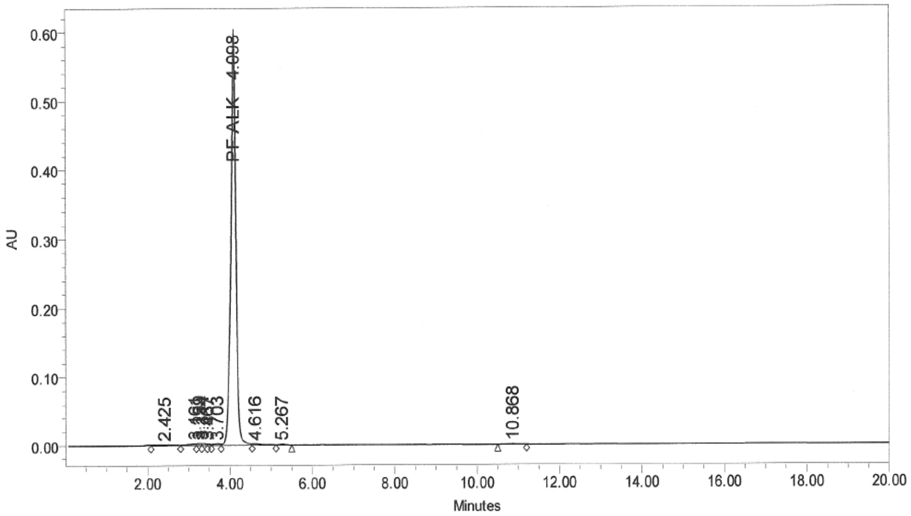

	Peak Name	RT (min)	Area (µV*sec)	% Area	Height (µV)	% Height	Amount	Units
1	Peak1	0.373						
2		2.425	43067	0.82	1339	0.22		
3		3.161	34562	0.66	2633	0.43		
4		3.259	22344	0.42	2923	0.47		
5		3.344	20744	0.39	2793	0.45		
6		3.467	11231	0.21	2141	0.35		
7		3.703	28625	0.54	2357	0.38		
8	PF ALK	4.098	5045411	95.84	600938	97.09	14.666	nmol
9		4.616	35618	0.68	1937	0.31		
10		5.267	11832	0.22	1267	0.20		

Supplementary Figure 15 (a) UV and (b) radioactivity trace; (c) HPLC UV trace of spiked sample. QC of [^{11}C]lorlatinib prepared on GE TracerLab FX_{FN} by 2-step radiosynthesis. (HPLC Column: Phenomenex Luna C-18 Analytical, 4.6 X 250 mm, 10 µm; Flow rate: 1 mL/min; Mobile Phase: ACN:0.1M AMF (60:40)).

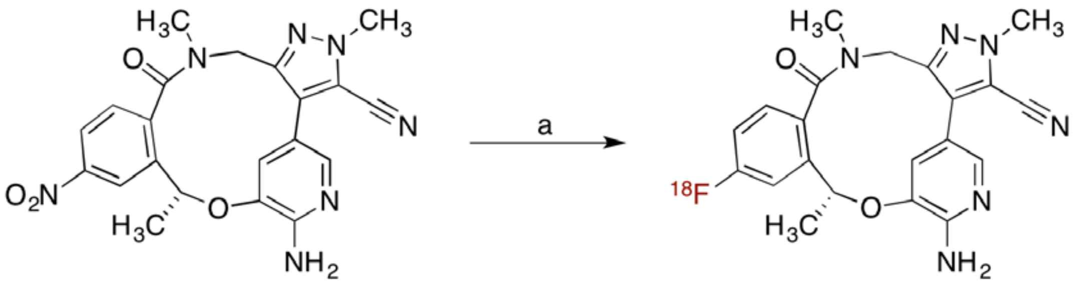

(*R*)-5 [18F](*R*)-1

Supplementary Figure 16. [^{18}F]Fluorination of Nitro precursor **(R)-5** using manual radiosynthesis

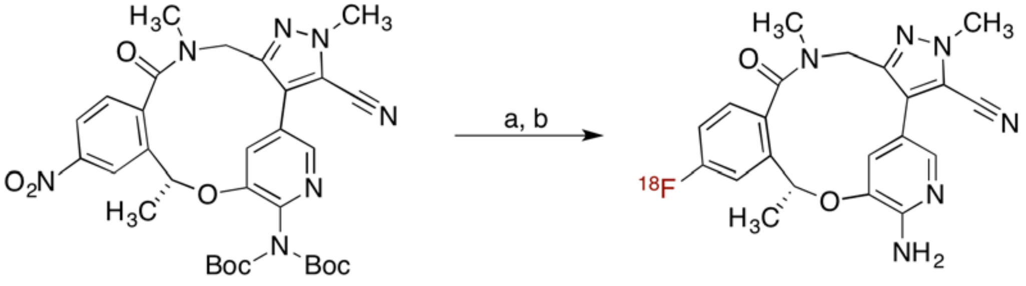

(*R*)-4 [18F](*R*)-1

Supplementary Figure 17. [^{18}F]Fluorination of protected Nitro precursor (*R*)-**4** using microfluidic radiosynthesis

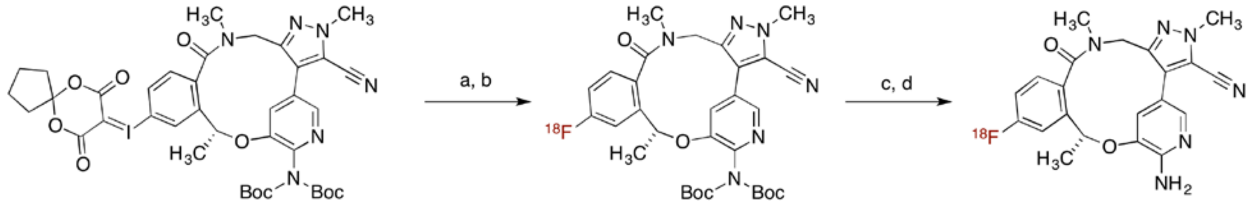

Supplementary Figure 18. Manual [^{18}F]fluorination of iodonium ylide precursor, ***(R)*-13**

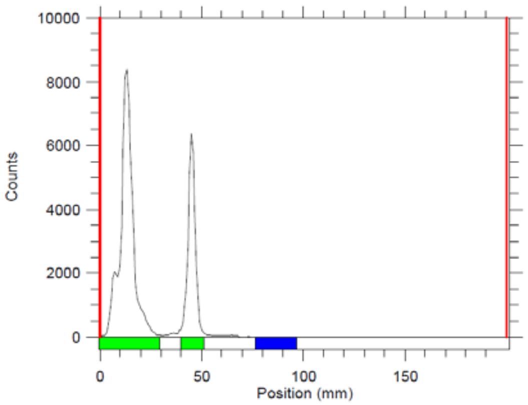

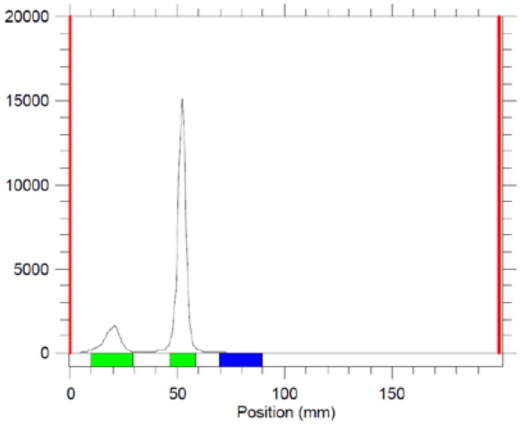

RadioTLC traces of crude reaction mixture (left) and [^{18}F]lorlatinib, **(R)-1**, after elution from a C18 SPE. Radiochemical conversions are provided in the table below (% RCC).

radioTLC, Silica Gel on Aluminum, with fluorescent indicator 254 nm
TLC plates: 5 X 10 cm,
Layer thickness: 200 µm
Particle size: 8.0-12.0 µm
Pore size: 60 Å medium pore diameter
Mobile Phase: EtOAc + 5% EtOH)

Yields of reactions of the manual radiosynthesis of [^{18}F]lorlatinib, **(R)-1**

**Run**	**1**	**2**	**3**	**mean**	**standard deviation**
**rTLC yield (%)**	36	31	22	27	7
**isolated yield (%)**	23	20	15	19	4

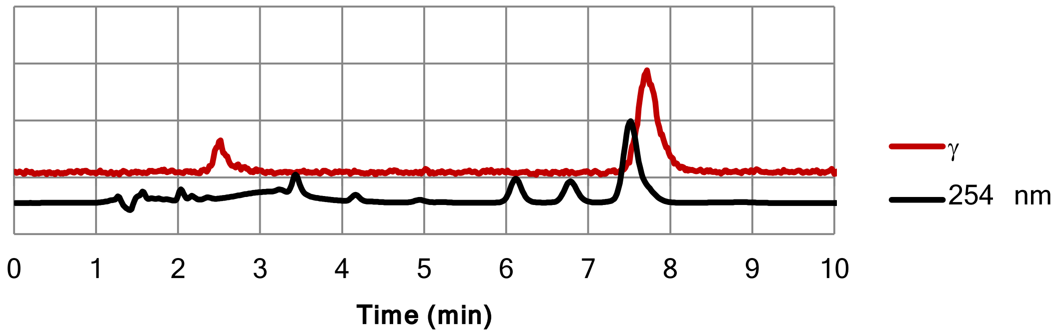

HPLC Trace of SPE purified [^{18}F]lorlatinib, **(R)-1**, with co-injection of cold standard
HPLC Column: Waters X-Bridge phenyl column, Analytical, 4.6 x 100 mm, 3.5 µm; Flow rate: 1 ml/min; Mobile Phase: ACN:0.1M AMF (30:70)

Supplementary Figure 19. Analysis of the manual radiosynthesis of [^{18}F]lorlatinib.

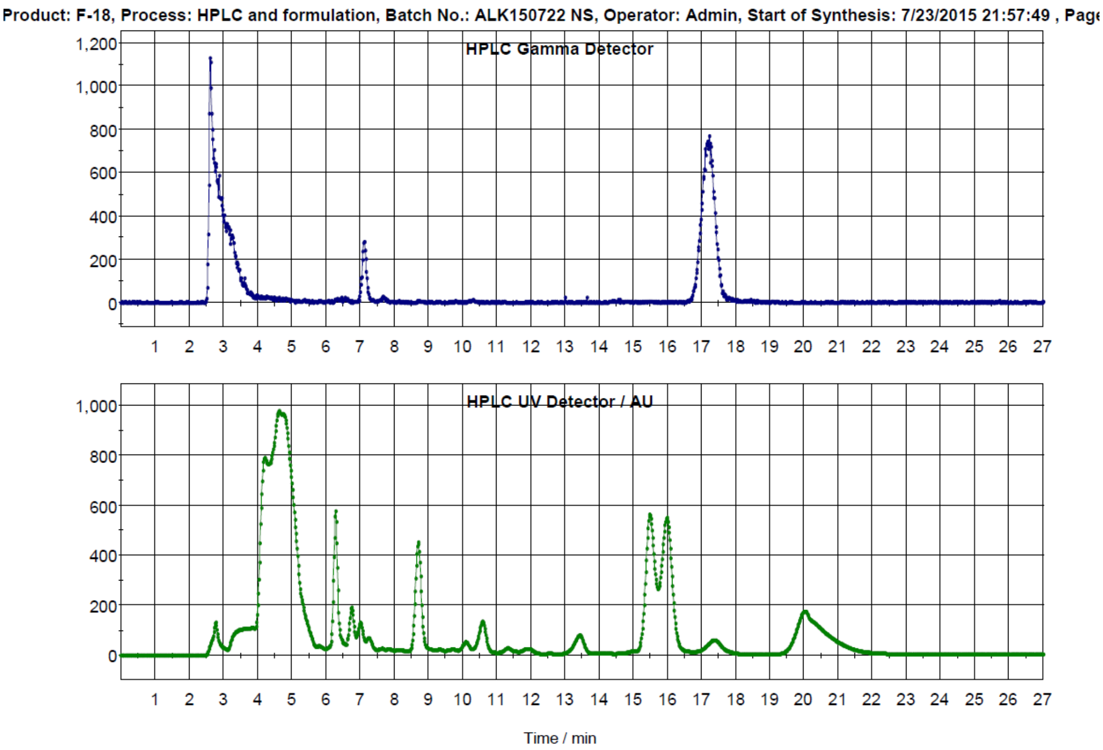

HPLC Trace of Isolation of Boc-protected by semi-preparative HPLC
HPLC Column: Phenomenex Luna C18 5u Semi-Prep column, 10 X 250 mm, 5 µm
HPLC Flow rate: 5 ml/min,
HPLC Mobile Phase: ACN:0.1M AMF (60:40)

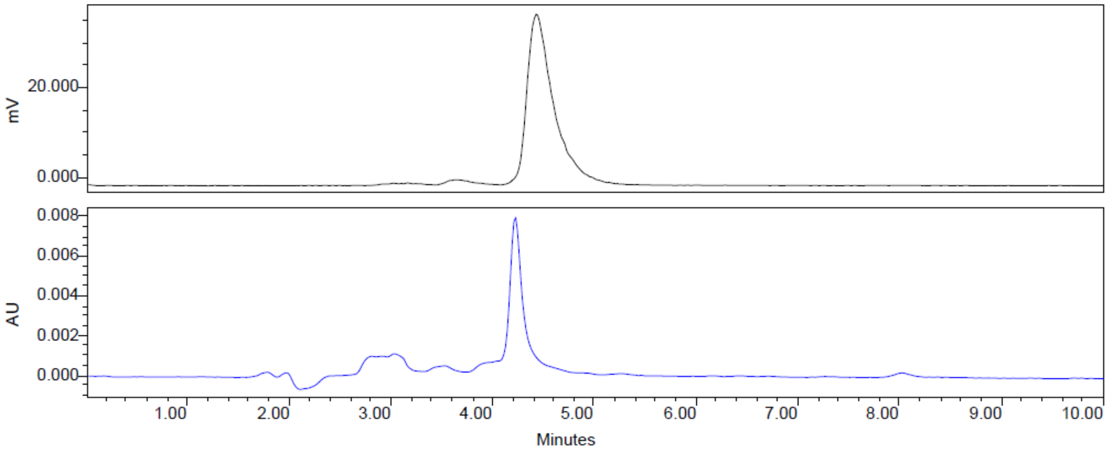

Analytical HPLC of [^{18}F]lorlatinib (*R*)-1 after elution from HLB cartridge and dilution with water (9 mL). Top trace = rad peak and bottom, blue trace = UV @ 254 nm. 50 µL injection. Radio concentration = 310 µCi/ mL @ 1:44 pm
HPLC Column: Phenomenex Luna C-18 Analytical, 4.6 X 250 mm, 10 µm
HPLC Flow rate: 1 ml/min,
HPLC Mobile Phase: ACN:0.1M AMF (60:40)

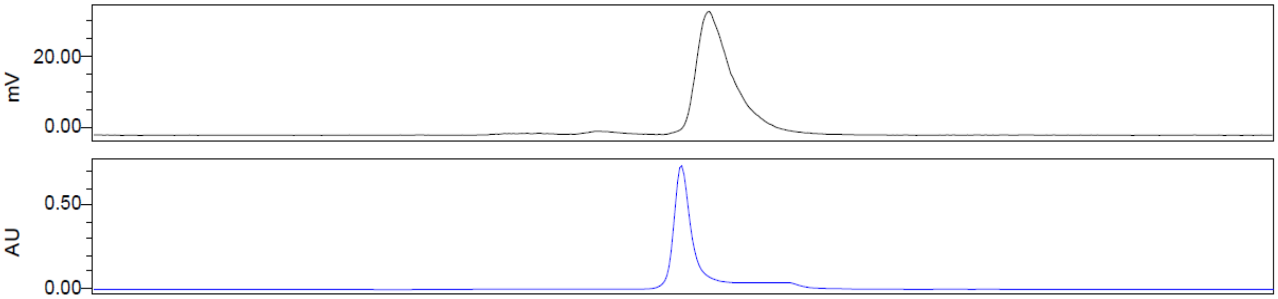

Coinjection of cold lorlatinib with [^{18}F]lorlatinib, (*R*)-1, on analytical HPLC after elution from HLB cartridge and dilution with water (9 mL). Top trace = rad peak and bottom, blue trace = UV @ 254 nm
HPLC Column: Phenomenex Luna C-18 Analytical, 4.6 X 250 mm, 10 µm
HPLC Flow rate: 1 ml/min,
HPLC Mobile Phase: ACN:0.1M AMF (60:40)

Supplementary Figure 20. Semi-preparative purification of the Boc protected labeled intermediate **(*R*)-8** and QC analysis of the final formulation of [^{18}F]lorlatinib **(*R*)-1**

Automation of [^{18}F]-lorlatinib labelling on FX_{FN} synthesis module and trapping on C18 Sep Pak.

The GE TracerLab FX_{FN} was cleaned using the standard cleaning method and then the following reagents were loaded to the respective vials:
Vial 1: tetraethylammonium bicarbonate, 3 mg in acetonitrile and water (1 mL, v/v 7:3)
Vial 3: Precursor **(R)-13**, (2 mg) was dissolved in anhydrous DMF (1 mL)
Vial 5: Anhydrous Acetonitrile (1 mL)
Vial 6: Sterile water for irrigation (2 mL)

Fluoride Trap: Sep-Pak® Light QMA Carbonate, prepared be flushing with TraceSELECT® Water (10 ml)
Intermediate Sep-Pak: Sep-Pak® Plus C-18, prepared by flushing with Ethanol (USP, 200 proof, 10 ml), Sterile water for irrigation (10 ml)
Reformulation Sep-Pak: Oasis® Plus HLB, prepared by flushing with Ethanol (USP, 200 proof, 10 ml), Sterile water for irrigation (10 ml)

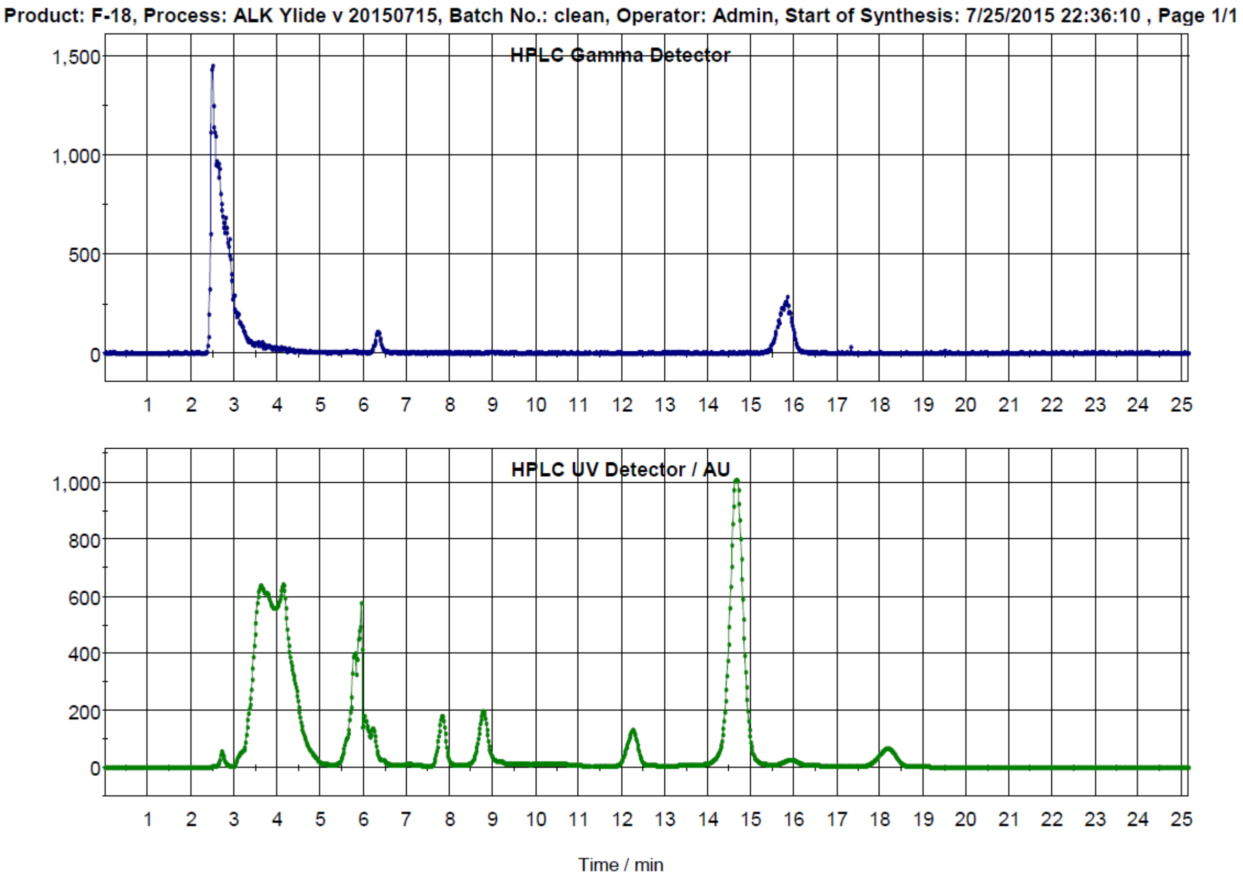

HPLC Trace of Isolation of Boc-protected intermediate, (*R*)-8, by semi-preparative HPLC
HPLC Column: Phenomenex Luna C18 5u Semi-Prep column, 10 x 250 mm, 5 µm
HPLC Flow rate: 5 mL/min,
HPLC Mobile Phase: ACN:0.1M AMF (60:40)

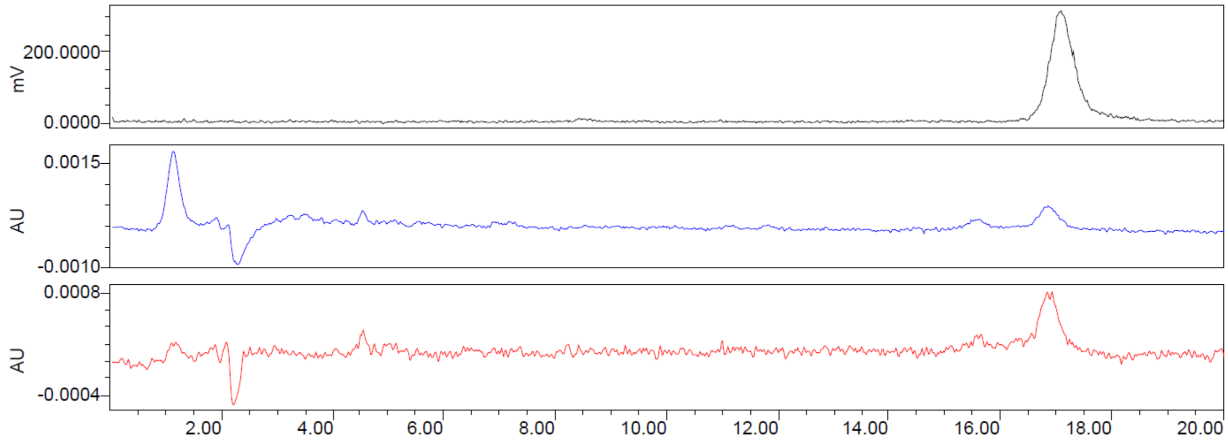

Analytical HPLC of eluted ethanol solution from C18-Sep-Pak. Top – Rad, Middle = UV @ 254nm and bottom = UV @ 290 nm

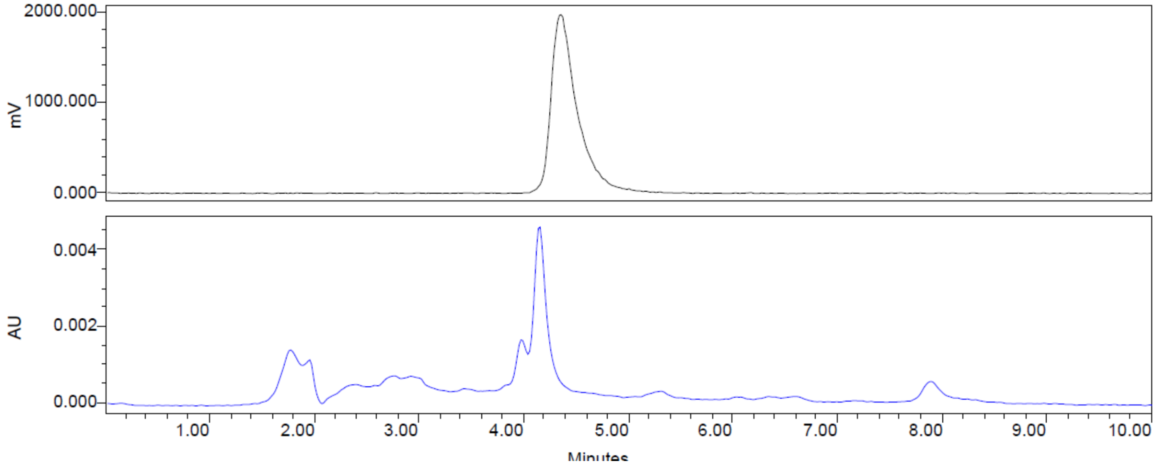

Analytical HPLC of [^{18}F]lorlatinib, (*R*)-1, after elution from HLB cartridge and dilution with water (9 mL). Top trace = rad peak and bottom, blue trace = UV @ 254 nm

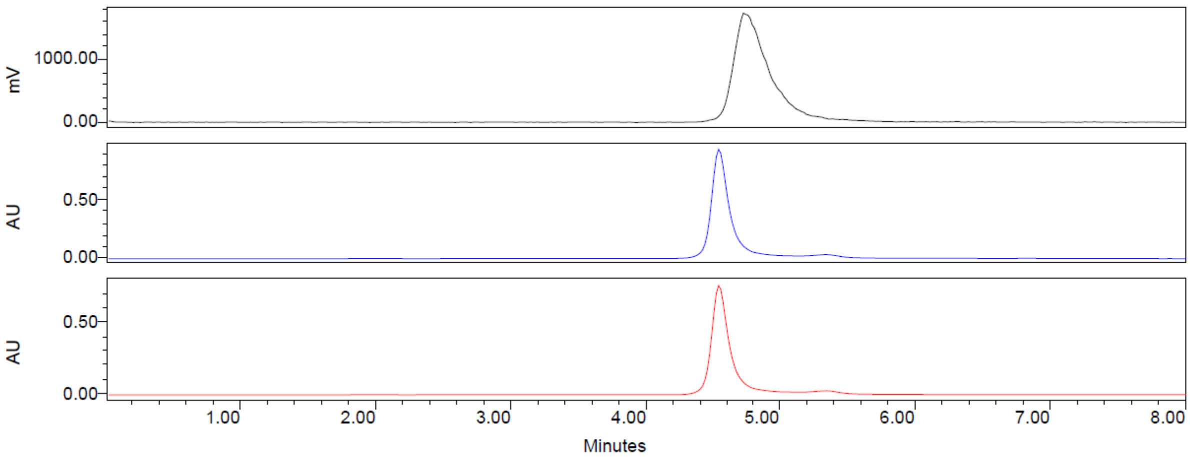

Co-injection of lorlatinib, (*R*)-1 on analytical HPLC after elution from HLB cartridge and dilution with water (9 mL). Top trace = rad peak and bottom, blue trace = UV @ 254 nm
HPLC Column: Phenomenex Luna C-18 Analytical, 4.6 X 250 mm, 10 µm
HPLC Flow rate: 1 ml/min,
HPLC Mobile Phase: ACN:0.1M AMF (60:40)imetry imaging studies

Supplementary Figure 21. Automated preparation and semi-preparative purification of the Boc protected labeled intermediate **(*R*)-8** and QC analysis of the final formulation of [^{18}F]lorlatinib **(*R*)-1**

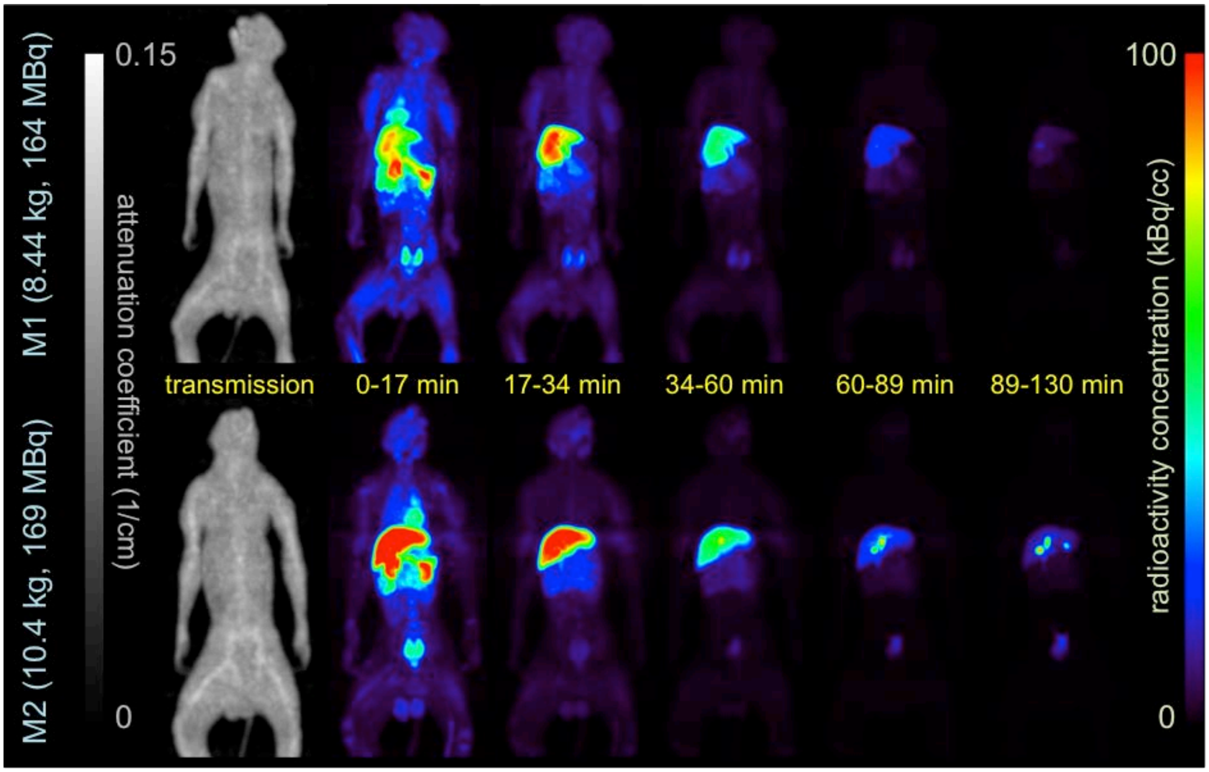

Supplementary Figure 22. Non-human primate imaging data with [^{11}C]lorlatinib. Whole body PET images acquired in each of two rhesus macaques (M1 and M2) over approximately two hours after i.v. injection of [^{11}C]lorlatinib. Data are presented in absolute radioactivity concentration without correction for radioactive decay.

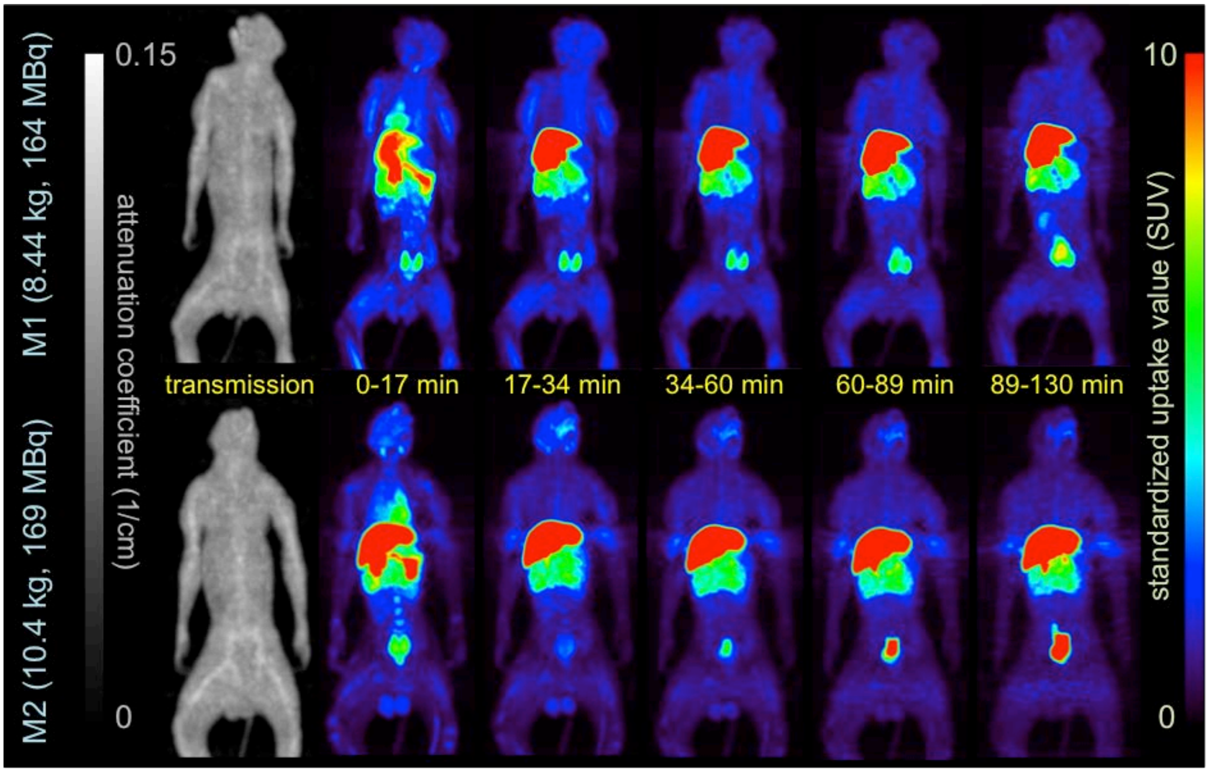

Supplementary Figure 23. Non-human primate imaging data (SUV) with [^{11}C]lorlatinib. Whole body PET images acquired in each of two rhesus macaques (M1 and M2) over approximately two hours after i.v. injection of [^{11}C]lorlatinib. Data are same as presented in previous figure, but in units of standardized uptake value (decay-corrected tracer concentration normalized by injected dose and body weight).

Supplementary Discussion

RADIATION DOSE ESTIMATES FOR ^{11}C—LABELLED LORLATINIB BASED ON DATA GATHERED IN PRIMATES

INTRODUCTION

Distributions of activity were determined in primates after injection of ^{11}C-labeled lorlatinib. Radioactivity levels were measured in various organs of the animals at 11 time points up to approximately 120 minutes post-injection. Radiation dose estimates were calculated based on these biokinetic data.

METHODS

Data were presented as PET image files. Counts were extracted from the image data using the MIPAV software[1]. Activity in each visualized organ and the total body were expressed as fractions of injected activity, normalizing the activity in the whole body at the earliest time point to be 100% of the administered activity. Resultant values of percent of injected activity per organ were fit using the SAAM II software[2]. Time integrals of activity[3] were entered into the OLINDA/EXM software[4], using the adult male model. No activity elimination was observed over this short imaging time. The number of disintegrations in the 'remainder of body' was assumed to that in total body minus the values in other organs of uptake.

RESULTS

The fitted metabolic model, number of disintegrations in the source organs, and organ doses are summarized below:

The fitted metabolic model was as follows (these values represent the % per organ):

Supplementary Table 1. Non-human primate dosimetry data with [^{11}C]lorlatinib

Animal #1

Organ	fraction	T-eff (min)	fraction	T-eff (min)
Total Body	1.000	20.32		
Liver	0.171	20.45	-0.134	0.069

The human radiation doses estimates are (adult male model):

Target Organ	mSv/MBq	rem/mCi
Adrenals	3.53E-03	1.30E-02
Brain	2.22E-03	8.23E-03
Breasts	2.26E-03	8.35E-03
Gallbladder Wall	4.42E-03	1.64E-02
LLI Wall	2.82E-03	1.04E-02
Small Intestine	3.06E-03	1.13E-02
Stomach Wall	2.98E-03	1.10E-02
ULI Wall	3.14E-03	1.16E-02
Heart Wall	3.09E-03	1.14E-02
Kidneys	3.17E-03	1.17E-02
Liver	1.49E-02	5.51E-02
Lungs	2.79E-03	1.03E-02
Muscle	2.55E-03	9.42E-03
Ovaries	2.94E-03	1.09E-02
Pancreas	3.52E-03	1.30E-02
Red Marrow	2.37E-03	8.78E-03
Osteogenic Cells	3.69E-03	1.37E-02
Skin	2.09E-03	7.72E-03
Spleen	2.76E-03	1.02E-02
Testes	2.40E-03	8.88E-03
Thymus	2.61E-03	9.67E-03
Thyroid	2.51E-03	9.31E-03
Urinary Bladder Wall	2.80E-03	1.03E-02
Uterus	2.95E-03	1.09E-02
Total Body	2.89E-03	1.07E-02
Effective Dose	3.23E-03	1.20E-02

Animal #2

Organ	fraction	T-eff (min)	fraction	T-eff (min)
Total Body	1.00	20.9		
Liver	0.33	19.8	-0.18	0.069

The human radiation doses estimates are (adult male model):

Target Organ	mSv/MBq	rem/mCi
Adrenals	3.97E-03	1.47E-02
Brain	1.89E-03	6.99E-03
Breasts	2.10E-03	7.78E-03
Gallbladder Wall	5.65E-03	2.09E-02
LLI Wall	2.43E-03	9.00E-03
Small Intestine	2.86E-03	1.06E-02
Stomach Wall	2.89E-03	1.07E-02
ULI Wall	3.09E-03	1.14E-02
Heart Wall	3.13E-03	1.16E-02
Kidneys	3.35E-03	1.24E-02
Liver	2.73E-02	1.01E-01
Lungs	2.81E-03	1.04E-02
Muscle	2.34E-03	8.67E-03
Ovaries	2.59E-03	9.60E-03
Pancreas	3.80E-03	1.41E-02
Red Marrow	2.24E-03	8.28E-03
Osteogenic Cells	3.28E-03	1.21E-02
Skin	1.87E-03	6.92E-03
Spleen	2.52E-03	9.32E-03
Testes	2.04E-03	7.56E-03
Thymus	2.36E-03	8.75E-03
Thyroid	2.16E-03	8.00E-03
Urinary Bladder Wall	2.41E-03	8.93E-03
Uterus	2.59E-03	9.58E-03
Total Body	3.01E-03	1.11E-02
Effective Dose	3.66E-03	1.35E-02

Average Doses:

Target Organ	mSv/MBq	rem/mCi
Adrenals	3.75E-03	1.39E-02
Brain	2.06E-03	7.61E-03
Breasts	2.18E-03	8.07E-03
Gallbladder Wall	5.04E-03	1.87E-02
LLI Wall	2.63E-03	9.70E-03
Small Intestine	2.96E-03	1.10E-02
Stomach Wall	2.94E-03	1.09E-02
ULI Wall	3.12E-03	1.15E-02
Heart Wall	3.11E-03	1.15E-02
Kidneys	3.26E-03	1.21E-02
Liver	2.11E-02	7.81E-02
Lungs	2.80E-03	1.04E-02
Muscle	2.45E-03	9.05E-03
Ovaries	2.77E-03	1.03E-02
Pancreas	3.66E-03	1.36E-02
Red Marrow	2.31E-03	8.53E-03
Osteogenic Cells	3.49E-03	1.29E-02
Skin	1.98E-03	7.32E-03
Spleen	2.64E-03	9.76E-03
Testes	2.22E-03	8.22E-03
Thymus	2.49E-03	9.21E-03
Thyroid	2.34E-03	8.66E-03
Urinary Bladder Wall	2.61E-03	9.62E-03
Uterus	2.77E-03	1.02E-02
Total Body	2.95E-03	1.09E-02
Effective Dose	3.45E-03	1.28E-02

CONCLUSIONS

Only data in liver and total body were evident in scans beyond the first time point. Several other organs were identifiable in the time zero image (kidneys, spleen, heart), but there was no apparent activity at the second time point, starting at about 8.5 minutes. Assigning time-activity integrals to these regions was not possible, and even a conservative approach would yield negligible numbers of disintegrations. Doses from any ^{11}C-labelled compound are generally low, due to the short physical half-life. With these data, the liver dose appears to be approximately 0.021 mSv/MBq, and the effective dose appears to be approximately 0.0035 mSv/MBq. As these estimates are based on animal data, studies in human subjects are needed to more firmly establish the dosimetry of this compound.

PRELIMINARY PET-CT IMAGING WITH ^{11}C—LABELLED LORLATINIB IN A TUMOUR BEARING MOUSE MODEL OF NSCLC

Human H3122 cells (EML4-ALK positive NSCLC cells) were cultured in RPMI containing 10% fetal bovine serum, and 1% Penicillin-Streptomycin at 37°C in a humid atmosphere containing 5% CO_2 and 95% air. A mouse model of human NSCLC was generated by injection of 5 x 10^6 cells in 0.2 ml of 1:1 (v/v) mixture of serum-free medium and Matrigel in the subcutaneous space of the athymic nude mice (n=9) using 25-gauge needle. Observation of a bulge under the skin was a sign of successful injection. Three weeks after tumor inoculation, the tumors reached 5-7 mm in diameter. Mice were divided into two groups: a total of 6 mice received 2.42 ± 0.68 mCi of [^{11}C]lorlatinib compound and 3 mice received mixture of [^{11}C]lorlatinib (2.18 ± 0.82 mCi) and 5 mg/kg of authentic non-radioactive lorlatinib (total injected volume < 300 ml). Dynamic PET/CT imaging was performed for 85 minutes after injection of the radiotracer using preclinical imaging scanner (Triumph II, Trifoil Imaging, Inc). Image analysis was performed with AMIDE (version 1.0.5) and the standard uptake value (SUV) and percentage of injected dose per gram of tumor tissue %ID/g (Mean ± SEM) was calculated.

The results show that the tumor uptake reached its plateau in approximately 30-60 minutes after injection of the [^{11}C]lorlatinib (with 2.2 – 2.37 %ID/g). Co-injection of [^{11}C]lorlatinib with non-radioactive lorlatinib resulted in significant decrease in the tumor uptake during the entire imaging course.

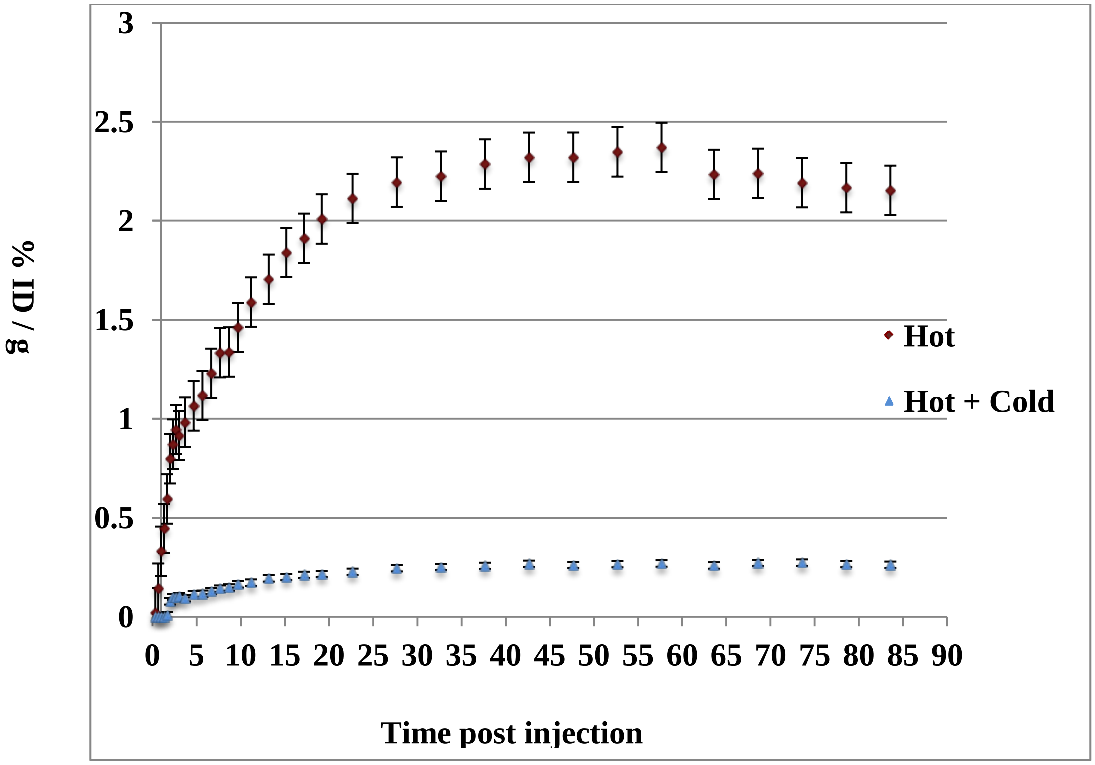

Supplementary Figure 24. Time-activity curve of uptake into tumor bearing mouse model. Baseline after injection of [^{11}C]lorlatinib (Hot) showing uptake reaching plateau over 2 SUV in the tumor vs. when co-injected with authentic lorlatinib (Hot + Cold) with uptake reaching a plateau at < 0.4 SUV over the course of 90 minutes.

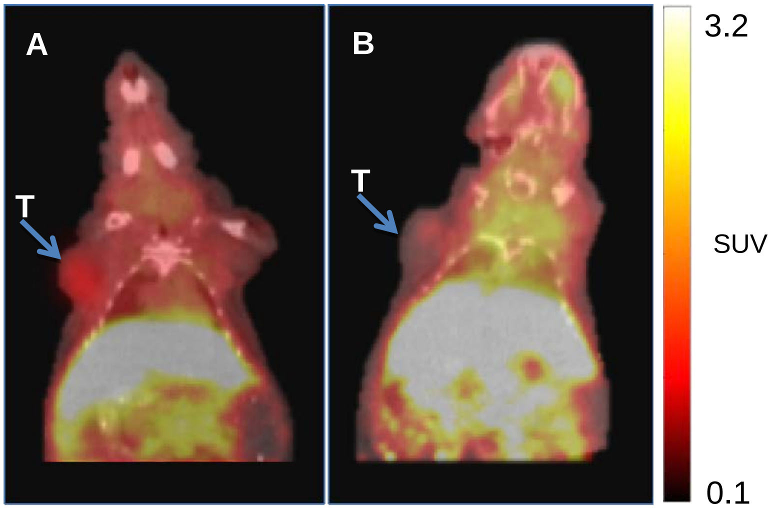

Supplementary Figure 25. PET-CT Imaging in Tumour Bearing Mice. Representative coronal PET-CT image showing tumor bearing mouse model at (A) baseline after injection of [^{11}C]lorlatinib vs. under blocking conditions (B) where the radiotracer was co-injected with authentic lorlatinib and a reduction in tumor uptake is observed.

Supplementary References

[1] McAuliffe MJ, Lalonde FM, McGarry D, Gandler W, Csaky K, Trus BL. Medical Image Processing, Analysis & Visualization In Clinical Research. IEEE COMPUTER-BASED MEDICAL SYSTEMS (CBMS) 2001, 381-386.

[2] Foster D. and Barrett P. Developing and testing integrated multicompartment models to describe a single-input multiple-output study using the SAAM II software system. In: Proc. Sixth International Radiopharmaceutical Dosimetry Symposium, Oak Ridge Institute for Science and Education, 1998

[3] Stabin MG and Siegel JA. Physical models and dose factors for use in internal dose assessment. Health Phys. 85, 294-310 (2003).

[4] Stabin MG, Sparks RB, and Crowe E. OLINDA/EXM: The Second-Generation Personal Computer Software for Internal Dose Assessment in Nuclear Medicine. J. Nucl. Med. 46, 1023-1027 (2005).
